# Developing knowledge‐based planning for gynaecological and rectal cancers: a clinical validation of RapidPlan^™^

**DOI:** 10.1002/jmrs.396

**Published:** 2020-05-25

**Authors:** Meegan Shepherd, Regina Bromley, Mark Stevens, Marita Morgia, Andrew Kneebone, George Hruby, John Atyeo, Thomas Eade

**Affiliations:** ^1^ Department of Radiation Oncology Northern Sydney Cancer Centre Royal North Shore Hospital Reserve Road, St Leonards NSW Australia; ^2^ Sydney Medical School University of Sydney Sydney Australia

**Keywords:** knowledge‐based planning, gynaecological neoplasms, rectal neoplasms, radiation oncology, decision making, service accessibility

## Abstract

**Introduction:**

To create and clinically validate knowledge‐based planning (KBP) models for gynaecologic (GYN) and rectal cancer patients. Assessment of ecologic generalisability and predictive validity of conventional planning versus single calculation KBP was reviewed against practical metrics of planning time (PT) and radiation oncologist plan preference.

**Method:**

Study cohorts were 34 and 42 consecutively treated GYN and rectal cancer patients dosimetrically archived within the centre’s research databank. For model training, structures and dose distributions from 22 and 32 GYN and rectal volumetric‐modulated arc therapy (VMAT) plans were used in RapidPlan™. Prescription doses ranged from 45 to 60Gy in 25 fractions using a simultaneous integrated boost to 2–4 targets and up to 9 organ‐at‐risk volumes. For model validation, 12 GYN and 10 rectal were independent of the archive and a single pass KBP VMAT plan was created. Each plan was evaluated against the archived treated plan under blinded conditions for radiation oncologist preference using standard dosimetric quality parameters.

**Results:**

All 22 plans generated in the KBP validation cohort met pre‐set GYN and rectal cancer dosimetric quality metrics. Fifty per cent of GYN plans and eighty per cent of rectal plans were judged superior to the manually optimised plans. KBP reduced PT considerably for both tumour sites.

**Conclusion:**

Single pass KBP for GYN and rectal cancer patients produced clinically acceptable treatment plans which were non‐inferior to conventionally optimised plans in 14 of 22 cases. Efficiencies captured by KBP will have predictable impacts on institutional workflows and resource allocation to facilitate adaptive planning.

## Graphical abstract

This study concerns the creation and clinical validation of knowledge-based planning (KBP) models for gynaecologic (GYN) and rectal cancer patients. Assessment involved the ecologic generalisability and predictive validity of conventional planning versus single calculation KBP in a limited KBP library against day-to-day practical metrics of planning time and radiation oncologist plan preference.

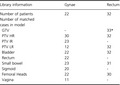

## Introduction

Inversely modulated planning processes for rectum and gynaecologic (GYN) sites are time‐consuming and labour intensive.^1^, ^2^ Despite sophisticated radiation therapy (RT) planning systems that automatically adjust and trade off dose to target and organ‐at‐risk (OAR) volumes, achieving the optimal patient plan often requires a high degree of dosimetrist intervention during planning optimisation.

With the introduction of population‐based screening programs, there has been a reduction in the caseload of locally advanced GYN and rectal cancers requiring neo‐adjuvant or definitive RT.^3^, ^4^ This poses resource implications, as dosimetrist experience and expertise can affect overall planning time and consistency.^2^, ^5^, ^6^, ^7^, ^8^, ^9^, ^10^, ^11^ The complexity of planning GYN and rectal sites is further compounded due to simultaneous integrated boost (SIB) techniques with multiple target volumes. These techniques require different fractionation schedules and need to consider internal target motion and the variability of individual patients relating to size, shape and location of OARs. This can create further resource issues for radiation oncology departments especially if patients require re‐planning for internal target volume changes or pelvic tilt.

Knowledge‐based planning (KBP) strategies have enabled these challenges to be addressed, with planning models being created from high‐quality archived treatment plans for specific anatomical sites.^10^, ^12^, ^13^, ^14^, ^15^, ^16^ RapidPlan^™^ (RP) from Varian Medical Systems (Palo Alto, USA) is one choice of commercial KBP software that creates these planning models. These models use dose‐volume histogram (DVH) estimation software to automatically determine optimisation objectives for new patient cases by comparing planning target volume (PTV) and OAR structures of plans loaded into the model. The predicted optimisation objectives drive the generation of a new plan with similar dosimetric quality to those in the planning model, to meet planning protocol requirements.^17^ The implementation, validation and training processes of KBP systems can be quite time‐consuming,^18^ and manufacturers require a minimum number of patient cases for model creation.^19^ This can be a barrier to the implementation of KBP systems in lower volume sites such as GYN and rectum, particularly in resource‐constrained environments.^18^

Knowledge‐based planning model predictability and reliability in producing acceptable plans is known to be relative to plan quality and the number of plans in a model across a range of treatment sites including cervix, prostate and head and neck cancer.^11^, ^20^, ^21^ The literature on KBP systems used on GYN and rectal cancers is minimal. Li et al^18^ report the use of KBP methods for plan quality control and consistency in GYN clinical trials. They reported the influence of high quality, carefully selected OAR sparing IMRT plans in RP DVH estimation models across two institutions. Such plans were superior for all DVH metrics to unfiltered models and manual planning. Hussein et al^11^ validated and benchmarked the use of RP in 37 single‐dose level cervix cancer patients in a single optimiser pass. They found in all cases PTV coverage was more conformal for RP when compared to conventionally optimised plans, and all OAR and normal tissue endpoints were lower for RP except bladder V30Gy. This demonstrates the ability of RP to successfully handle the variations that are presented in pelvic cancer patient planning. Previously reported patient case numbers for GYN and rectal KBP model studies ranged from 37 to 86.^11^, ^13^, ^20^ There is minimal information on the effectiveness of small patient case numbers for KBP models in GYN and rectal sites planned with volumetric‐modulated arc therapy (VMAT).

This study focuses on the generalisability and predictive validity of KBP in GYN and rectal sites with RP models using small patient numbers to create clinically acceptable VMAT plans. Additionally, the study records planning time (PT) constraints and radiation oncologist plan preference (ROPP) between RP generated plans and previously treated conventionally optimised plans (CP).

## Methods

### Patient selection

Data used in this study were accessed from our research databank (Northern Sydney Local Health District (NSLHD) ethics reference: RESP/15/255). These patients had undergone external beam RT at our centre for either GYN or rectal cancer using department protocols.

Between January 2014 and March 2017, 36 consecutive patients with uterine cervix, endometrium or vulvar cancer and 42 patients with rectal cancer were enrolled for external beam radiotherapy in our centre. All patients were scanned with a slice thickness of 2mm on a Phillips (Brilliance Big Bore) computed tomography (CT) scanner in the supine position with a knee block and ankle stock immobilisation. Following CT, the images were transferred to Eclipse treatment planning system (TPS) (version 13.6.23) for contouring and planning.

For patients having GYN treatment, the target volumes included the whole uterus, cervix and part of the vagina depending on the lower extent of the tumour, the paracervical, parametrium and uterosacral regions, as well as the common iliac, external iliac, internal iliac and obturator lymph nodes. The (PTV) was defined around the clinical target volume (CTV) contoured by the radiation oncologist (RO) with a margin of 7mm. In patients receiving rectum treatment, the target volume included the gross disease (primary lesion), clinical and subclinical disease in nodal groups around the gross disease, pre‐sacral space, internal iliac lymph nodes and mesorectum, and in some cases the ischiorectal fossa, obturator nodes, anastomosis, perineum and other pelvic organs. The PTV was defined around the CTV contoured by the RO with a margin of 7mm.

Whole pelvic VMAT simultaneous integrated boost (SIB) plans were created using 6–10 MV in two or three arcs to deliver prescription doses that were dependent on disease risk level. For GYN sites, a doses of 54–55Gy in 25 fractions to the areas of high risk (PTV_HR), 50Gy in 25 fractions to the areas of intermediate risk (PTV_IR) and 45Gy in 25 fractions to the low‐risk prophylactic lymph node volumes (PTV_LR). For the rectum sites, doses of 54Gy in 25 fractions, 50Gy in 25 fractions and 45Gy in 25 fractions were delivered to areas of very high (PTV_VHR), high (PTV_HR), and low (PTV_LR) risk, respectively.

The OARs for both models included the bladder, sigmoid colon, small bowel, anal canal, external genitalia and femoral heads. Additional GYN OARs included rectum and iliac bone marrow.

### Knowledge‐based planning model

The KBP software determines optimisation objectives from the patient geometry and previous dosimetry contained in an archived set of existing patient cases within the library. For the GYN and rectum RP models, a consecutive cohort of 22 and 32 patient plans and their contours were exported. Patient numbers were chosen as they were the minimum number of cases available to create a RP model.^19^ The remaining 6 GYN and 10 rectum cases were used for validation of the models. The information from existing treatment plans was used to automate the planning optimisation process for both sites.

The primary site and prescription dose for patients included in the GYN model are shown in Table 1. Targets for patients with dose fractionations of 55Gy included high‐, intermediate‐ and low‐risk volumes with both primary involvement and nodal involvement. For patients with doses less than or equal to 50Gy, intermediate‐ and low‐risk target volumes for both primary and nodal malignancies accounted for the remainder of the GYN model. For the rectum model, all planning target volumes included PTV high and low risk, with one case also having a very high‐risk volume.

**Table 1:** GYN Model patient specifications (n = 22): primary site and prescription dose

Primary site	Dose/Fractionation (Gy/Fx)
Cervix (6) Endometrium (1) Vagina (1) Vulva (1)	55/25
Endometrium (5) Cervix (1) Vaginal (1)	50/25
Cervix (1)	47.5/25
Uterus (3) Endometrium (1) Vaginal (1)	45/25

Abbreviation: GYN = gynaecologic.

In Eclipse and RapidPlan version 13.6.23, RP allows the user to define the standard optimisation objectives to be used for up to three target volumes. For the rectum model, standard target optimisation objectives were applied to the GTV, PTV_HR and PTV_LR volumes. Due to the limitation on the number of target volumes, in the GYN model the PTV_HR volumes, both primary and nodal volumes, were combined into the one model structure named PTV_HR.

The model data were trained for each of the models. This process generates mathematical parameters through analysis of the geometric and dosimetric statistics of the PTVs and OARs from the uploaded plans which are used for DVH estimations. The trained models were then exported to model analytics, providing an indication of potential outlier plans, contours and DVHs that can decrease the integrity and reliability of the model.^17^ The software allows for plans to be removed entirely or just outlier contours to be removed before the trained model is created. Each outlier identified by the statistical processes in Model Analytics for both GYN and rectum models was scrutinised by the dosimetrist responsible for creating and testing the models. Outlier assessment was for large values only, as exclusion of all outliers could potentially diminish the quantity of contours needed to create a valid model. After outlier plan assessment, it was determined that no whole plans or contours would be removed from either model. Table 2 details the number of patients, targets and OAR library volumes for the GYN and rectum models.

**Table 2:** GYN & Rectum KBP library information: Target and OAR volumes

Library information	Gynae	Rectum
Number of patients	22	32
Number of matched cases in model		
GTV	‐	33*
PTV HR	30	32
PTV IR	23	‐
PTV LR	12	32
Bladder	22	32
Rectum	22	‐
Small bowel	23	31
Sigmoid	20	‐
Femoral Heads	22	30
Vagina	11	‐

Abbreviations: GTV = gross tumour volume; GYN = gynaecologic; HR = high risk; IR = intermediate risk; KBP = knowledge‐based planning; LR = low risk; PTV = planning target volume.

* Multiple GTV’s for one patient

When creating a KBP model, the user defines the OAR volumes for the model that will determine optimisation objectives for future patients using a line function. A line function is created from the DVH data of all patient cases in the KBP model. For each OAR, a minimum of 20 structures are needed in a model for the DVH estimations software to generate the OAR line objective.

Upper, lower and line objectives and priorities were created in model configuration for each target volume and OAR to achieve the standard GYN and rectum treatment protocols for target volumes (Supporting Information) and OARs (Tables 3 and 4). For the bladder, rectum, sigmoid and small bowel, where the accepted dose was influenced by geometric factors, a generated line objective and priority was added. According to our departmental planning protocol, additional dose modification structures including OARs 10mm away from the target volumes such as rectum_1cm_out (GYN model) and bladder_1cm_out (both models) were added and assigned optimisation objectives and priorities for greater reduction in dose received. The 1cm out Boolean structures were common to both CP and KBP. Additionally, fixed maximum objectives were placed on the OARs to avoid hot spots. For dose optimisation, PTV structures were separated with the use of the Boolean tool. After generating optimising values, the optimising priorities were added to these structures manually to ensure adequate coverage and give the planner more control over the location of hot spots. A normal tissue objective (NTO) was used to create a fall‐off from the target structures, reducing dose to surrounding tissue.

**Table 3:** GYN treatment planning protocol: Organs at Risk

OAR structures	Objective	Minor deviation
Bladder	V45 < 35%	V50 < 35%
Rectum	V30 < 60%	V50 < 35%
Sigmoid	V30 < 60%	V50 < 35%
Femoral heads	V30 < 15%	V30 < 20%
Kidneys	V20 < 32% V28 < 20% Mean < 15–18Gy	V30 < 25%
Pelvic bone marrow	V15 < 75% V10 < 90%	V15 < 80% V10 < 95%
Small bowel	V40 < 30%	
1. Peritoneal cavity (Peritoneal volume edited off PTV)	V40 < 200–300cc V45 < 100cc	V45 < 195cc
2. Individual loops	V15 < 120cc	

Abbreviations: GYN = gynaecologic; OAR = organ at risk, PTV = planning target volume.

**Table 4:** Rectum treatment planning protocol: Organs at Risk

OAR structures	Objective	Minor deviation
Small bowel in PTV	<102% (51Gy)	
Small bowel sub‐PTV	1cc < 50Gy	100cc < 40Gy
Femoral heads	V30 < 15%	
Anal canal (outside PTV)	95% isodose within 2mm of PTVLD	
Bladder out	V50Gy < 10%	V50Gy < 20%
(Bladder sub (CTVHD + CTVLD)	V40Gy < 25%	V40Gy < 35%
External genitalia	V20Gy < 10%	V20Gy < 20%
Peripheral tissue	Max < 40Gy	

Abbreviations: CTV = clinical target volume; HD = high dose; LD = low dose; OAR = organ at risk; PTV = planning target volume.

Twelve and ten previously treated GYN and rectum patients, respectively, were optimised using the relevant models. These patient cases were consecutively selected from the initial cohort and were not included in the creation of the models. All plans were optimised and calculated using version 13.6.23 photon optimiser and AAA dose algorithm. The KBP VMAT plans were created using a single pass through the photon optimiser with the same isocenter, collimator angles and field sizes as the original clinically treated plans, and no intervention from the planner.

### Clinical comparison: knowledge‐based planning versus conventional endpoints

Twenty‐four GYN VMAT plans (twelve KBP plans and twelve CP) were assessed by two ROs with expertise in GYN cancer, and twenty rectal VMAT plans (ten KBP, ten CP) were assessed by one RO with expertise in rectal cancer. All ROs undertaking plan assessment had prescribed the original plans used in creating each of the KBP models. The plans were assessed for target dose coverage and dose to OARs according to the department planning protocols for each site. All ROs were blinded to the optimisation technique used, with plans randomly assigned A or B for review. When conducting the plan evaluation, each RO was required to assess the clinical acceptability of each plan based on departmental planning protocols (Supporting Information, and Tables 3 and 4) as well as nominate a preferred plan for each patient and provide a rationale for their preference. Plan assessment was done via dose wash distribution, DVH and clinical protocol review.

### Time comparison: knowledge‐based planning versus conventional planning

Each KBP‐generated plan was optimised in a single pass with no planner intervention. This was compared to the number of optimisations required for the RT planner to generate clinically acceptable plans for eight CP, four GYN and four rectum plans of similar complexity to those in the validation cohort. These data were collected from an in‐house planning timing study in 2016/2017. None of the eight plans were included in the KBP models or validation cohort.

## Results

All 22 KBP‐generated plans (12 GYN and 10 rectum) using one pass through the optimiser were deemed clinically acceptable by all ROs with no major protocol violations for target coverage or OAR doses.

Eight of ten rectum KBP plans were selected by radiation oncologists as superior to the conventionally optimised treatment plans based on the similarity of target coverage and lower OAR doses. Table 5 details the preferred plan and rationale in the rectum validation cohort. The two KBP plans (patients 1 and 10) considered inferior were due to small areas of higher dose in the small bowel and reduced perceived homogeneity of dose by prescribing radiation oncologists when compared to the treated plan, although both were considered clinically acceptable.

**Table 5:** Rectum KBP validation: preferred plan and rationale.

Patient	Preferred	Primary rationale	Secondary rationale
1	Conventional Plan	Target conformity and homogeneity	Reduced hot spots in PTV LD
2	RapidPlan	Superior OAR sparing (small bowel)	
3	RapidPlan	Superior OAR sparing (small bowel and bladder)	
4	RapidPlan	Improved hot spot location (near GTV) and target homogeneity	Superior OAR sparing (bladder)
5	RapidPlan	Superior OAR sparing (small bowel and bladder)	Superior PTV coverage
6	RapidPlan	Superior OAR sparing (small bowel and bladder)	
7	RapidPlan	Superior OAR sparing (small bowel and bladder)	
8	RapidPlan	Superior OAR sparing (small bowel and bladder)	
9	RapidPlan	Superior OAR sparing (bladder)	
10	Conventional Plan	Target conformity	Target coverage at pre‐sacral space

Abbreviations: KBP = knowledge‐based planning; LD = low dose; OAR = organ at risk; PTV = planning target volume.

Six of the twelve GYN KBP plans were selected as superior to the original clinically treated plan based on ROPP. Table 6 details each patient in the GYN KBP validation cohort, the preferred plan and rationale. In all decisions, expert radiation oncologist agreed on the preferred plan as well as primary and secondary rationale. The six preferred GYN KBP plans were considered superior to the CP for target coverage, OAR doses or both. For the six KBP plans considered inferior to the CP, but still clinically acceptable, target dose inhomogeneity and decreased conformity, leading to higher dose to OARs, were cited as the reasons for RO preference.

**Table 6:** GYN KBP validation: preferred plan and rationale

Patients	Primary site	Preferred	Primary rationale	Secondary Rationale
1	Endometrium	RapidPlan	Target conformity	
2	Endometrium	RapidPlan	Superior OAR sparing (rectum & bladder)	
3	Cervix, Nodes	Conventional plan	Superior OAR sparing (rectum & bone marrow)	
4	Cervix, Nodes	Conventional plan	Target conformity	
5	Cervix, Nodes	Conventional plan	Target homogeneity	Superior OAR sparing (bladder and small bowel)
6	Cervix, Nodes	RapidPlan	Target conformity	
7	Endometrium	RapidPlan	Target conformity	Superior OAR sparing (bladder)
8	Cervix, Nodes	RapidPlan	Superior OAR sparing (bladder)	
9	Cervix, Nodes	Conventional plan	Target conformity	Superior OAR sparing (bone marrow & bladder)
10	Uterus	Conventional plan	Target homogeneity	Superior OAR sparing (sigmoid & rectum)
11	Uterus	RapidPlan	Superior OAR sparing (bladder)	
12	Endometrium	Conventional plan	Target homogeneity	

Abbreviations: GYN = gynaecologic; KBP = knowledge‐based planning; OAR = organ at risk.

### Planning time

Each KBP plan was optimised only once with no planner intervention taking approximately 25 minutes. In comparison, our in‐house timing study showed conventionally optimised GYN and rectum planning on average required a total of 14 (range 6–17) and 19 (range 10–27) optimisations, respectively. With current departmental planning configuration, this equates to 403 minutes (range 180–640) and 485 minutes (range 200–750) of optimisation time for GYN and rectum plans, respectively.

## Discussion

We have demonstrated that using KBP models with small patient case numbers to generate new GYN and rectum VMAT treatment plans is more efficient than conventionally optimised planning. Before the use of KBP at our centre, GYN and rectum sites required on average 14 and 19 optimisations and 6.7 and 8.1 hours of optimisation time, respectively, to achieve a clinically acceptable plan. This is consistent with Hussein et al^11^ and Wang et al^22^ who describe high levels of planner intervention to ensure a truly optimal plan during conventional trial‐and‐error inverse planning, due to suboptimal or conflicting optimisation constraints. With the use of KBP for GYN and rectal sites, we are able to consistently achieve clinically acceptable plans in a single optimisation which has shown real‐world (i.e. ecologically valid) gains such as reduced planning time, and decreased burden on the optimisation software and planning resources. This is similar to the findings of Wu et al^23^ who report that a typical RP VMAT rectum is finished in about 30 minutes without any interactive objective adjustment.

In this present study, the number of patient cases in each RP model was considered relatively small with 22 and 32 in the GYN and rectum models, respectively, compared to other KBP models quoted in the literature such as Hussein et al^11^ (37 cervix), Lian et al^13^ (86 cervix), Wang et al^22^ (81 Rectum), Wu et al^23^ (80 rectum), Chanyavanich et al^21^ (100 prostate) and Good et al^14^ (132 prostate). In this study, small case numbers in each of the KBP models introduced variability of primary sites and prescription doses particularly in the GYN model. However, successful plan generation from clinically acceptable plans for the validation cohort in a single optimisation is encouraging. This optimisation tool has positive implications for radiation oncology departments with smaller patient throughput who wish to adopt KBP. Wang et al^22^ detail that incorporating new and improved patient plans into the original KBP training library can improve model output for the previous and upcoming plans. With previous literature indicating the success of KBP‐generated plans being dependent on the quality of the plans in the KBP model^11^, ^24^, ^25^, this remains an area for future analysis.

All 22 KBP‐generated plans were deemed clinically acceptable from a single optimisation indicating good plan quality in both models. However, 50% of the GYN and 20% of the rectum KBP‐generated plans were considered inferior to conventional optimised methods, citing unfavourable dosimetric factors such as target dose inhomogeneity and decreased conformity, leading to higher doses to OARs. This is similar to the validation of 30 VMAT rectum cases by Wang et al^22^ whereby not all KBP‐generated plans yielded better dosimetric outcomes when compared to conventionally optimised plans. During blinded review by radiation oncologists, patient case number 2 in the GYN validation cohort yielded lower doses to OARs with similar target coverage using KBP when compared to manual methods. This may have been due to the use of a line objective with multiple optimiser points on the DVH for OARs such as bladder, rectum and sigmoid. In light of this, any single optimisation KBP‐generated case needs careful plan evaluation by the dosimetrist and RO, and if found to be dosimetrically suboptimal, plan refinement with patient‐specific adjustment to objectives and priorities with re‐optimisation should be considered irrespective of additional planning time. KBP‐generated plans in this study were only optimised once, and it is unknown that if inferiorly preferenced plans were optimised multiple times, whether they would have yielded a better plan for target and OAR doses than the original or manually generated plan.

An important aspect of this study is the applicability of the KBP models to various primary sites, and their complexity of target volumes and prescription doses. SIB techniques with multiple dose level prescriptions are common practice in pelvic RT. Variation in the site models may have attributed to the difference in success rates between the GYN and rectal validation of RP. The GYN model holds cases with five primary sites, across four dose levels when compared to the rectal model, with all but one case matched to the primary site and target doses. This poses future questions for the creation of single site and dose level KBP models in GYN RT. In this instance, targets were often combined or normalised to a single‐dose level for KBP optimisation.^11^, ^14^ For the GYN model, PTV_HR primary and nodal volumes were combined due to the maximum of three target volumes available in the RP version used in this study. Combining targets and dose prescription variation did not degrade the KBP model’s ability to produce a clinically acceptable plan. Version 15.6 of RP allows for up to ten targets and will allow for targets to be calculated as independent volumes in the future. A future study will evaluate the differences between model versions.

The use of KBP can further help dosimetrists of any skill and experience level to create clinically acceptable plans for rectum and GYN sites. By means of machine learning, RP applies a department’s collective dynamic planning experience as a strong baseline upon which to build individualised plans, indeed the majority, producing a clinically acceptable plan in a single iteration. Previous literature details the heavy dependence on the experience and skill of the treatment planner in the planning time required to produce clinically acceptable plans. ^17^ In this study, across both GYN and rectum sites, radiation oncologist plan preference was not dependent upon the number of optimisations or the method used for optimisation. Reducing the number of optimisations and planning time has implications for departmental rostering and workflow, as initial KBP optimisations can be optimised by a dosimetrist of any experience level.

One limitation in this study is that plan quality assessment between the KBP and the CP was completed via non‐statistical methods including dose wash review, electronic clinical protocol assessment and DVH comparison, which is the current departmental method for plan assessment. This differs from the literature on KBP effectiveness, which standardly compares target doses and organ a risk exposure using a statistical approach.^11^ Future directions include an updated departmental planning timing study and improvement of initial KBP models with statistical assessment of KBP and CP. Re‐evaluation and development of departmental KBP models will come from the addition of further patients to the libraries and an upgrade of Varian Rapid Plan to version 15.6. This will ensure a greater representation of the anatomical variation of patients with more target volumes within the new models to predict optimal optimisation objectives for OARs for future patients.

By incorporating RP and the development of KBP models into clinical planning processes, increased clinical efficiencies are introduced through reduced optimisations without affecting dosimetric plan quality. These efficiencies are expected irrespective of treatment site, dosimetrist experience level or treatment site. In the future, dosimetrists can create clinically acceptable plans in a single pass through the optimiser using RP. RP will also be a useful time‐saving tool when moving towards an adaptive planning or plan of the day approach, particularly in GYN sites where internal target motion impacts on target volume geography.

## Conclusion

Using small case numbers in the development of KBP models, radiation therapy planning using RapidPlan^™^ for gynaecologic and rectal techniques was successful in generating clinically acceptable plans in a single pass through the optimisation software. In reducing the need for multiple, time‐consuming iterations, there are beneficial implications for department workflow, adaptive planning and automated quality assurance.

## Supporting information


**Supporting Information I**. GYN treatment planning protocol: target volumes.
**Supporting Information II**. Rectum treatment planning protocol: target volumes.
