# Supplementary material for: Developing knowledge‐based planning for gynaecological and rectal cancers: a clinical validation of RapidPlan™
Source: J Med Radiat Sci. 2020 May 25;67(3):217–24. doi: 10.1002/jmrs.396 (PMC7476182; doi:10.1002/jmrs.396)
Supplement: Supplementary file 1 — Supporting Information I. GYN treatment planning protocol: target volumes. Supporting Information II. Rectum treatment planning protocol: target volumes. [file JMRS-67-217-s001.docx]

Supporting Information I: GYN treatment planning protocol: target volumes

| **Volume** | **Objective** |
| --- | --- |
| GTV_P  GTV_N | 98% vol = 100% dose  [55Gy] |
| CTV_HR_P  CTV_HR_N | 98% vol = 98% dose  [53.9Gy] |
| PTV_HR_P  PTV_HR_N | 98% vol = 95% dose  [52.25Gy] |
| CTV_IR_P  CTV_IR_N | 98% vol = 98% dose  [49Gy] |
| PTV_IR_P  PTV_IR_N | 98% vol = 95% dose  [47.5Gy] |
| CTV_LR_P | 98% vol = 98% dose  [44.1Gy] |
| CTV_LR_N | 98% vol = 100% dose  [45Gy] |
| PTV_LR_P | 98% vol = 95% dose  [42.75Gy] |
| PTV_LR_N | 98% vol = 100% dose  [45Gy] |

*Abbreviations:* GYN = gynaecologic; GTV = gross tumour volume; PTV = planning target volume; CTV = clinical target volume; P = primary; N = nodes; HR = high risk; IR = intermediate risk; LR = low risk

Supporting Information II: Rectum treatment planning protocol: target volumes

| **Volume** | **Objective** |
| --- | --- |
| PTV VHD | 98%vol = 54Gy |
|  | Max<107% [57.76Gy] |
| GTV + 1cm | 100% vol = 100% dose  [50Gy] |
|  | Max<110% [55Gy] |
| CTV HD | 98% vol = 100% dose  [50Gy] |
| PTV HD | 95% vol = 100% dose  [50Gy] |
|  | Max<107% [53.5Gy] |
| CTVLD | 98% vol = 100% dose  [45Gy] |
| PTVLD | 95% vol = 100% dose  [45Gy] |
|  | Max < 107% [48.2Gy] |

*Abbreviations:* PTV = planning target volume; GTV = gross tumour volume; CTV = clinical target volume; VHD = very high dose; HD = high dose; LD = low dose
